# Meeting Report: WHO Workshop on modelling global mortality and aetiology estimates of enteric pathogens in children under five. Cape Town, 28–29th November 2018

**DOI:** 10.1016/j.vaccine.2020.01.054

**Published:** 2020-06-26

**Authors:** H.J. Prudden, M. Hasso-Agopsowicz, R.E. Black, C. Troeger, R.C. Reiner, R.F. Breiman, M. Jit, G. Kang, L. Lamberti, C.F. Lanata, B.A. Lopman, W. Ndifon, V.E. Pitzer, J.A. Platts-Mills, M.S. Riddle, P.G. Smith, R. Hutubessy, B. Giersing

**Affiliations:** a Initiative for Vaccine Research, World Health Organisation, CH-1211 Geneva, Switzerland; b Johns Hopkins Bloomberg School of Public Health, Baltimore, MD 21205, USA; c Institute for Health Metrics and Evaluation, University of Washington, Seattle, WA 98121, USA; d Global Health Institute, Emory University, Atlanta, GA, USA; e Department of Infectious Disease Epidemiology, London School of Hygiene & Tropical Medicine, United Kingdom; f Modelling and Economics Unit, National Infections Service, Public Health England, United Kingdom; g School of Public Health, University of Hong Kong, Hong Kong; h Translational Health Science and Technology Institute, Faridabad, India; i Bill & Melinda Gates Foundation, Seattle, WA, USA; j Instituto de Investigacion Nutricional, Lima, Peru; k Department of Pediatrics, School of Medicine, Vanderbilt University, Nashville, TN 37027, USA; l African Institute for Mathematical Sciences, Cape Town, South Africa; m Department of Epidemiology and Microbial Diseases, Yale School of Public Health, Yale University, New Haven, CT, USA; n Division of Infectious Diseases & International Health, University of Virginia, Charlottesville, VA 22908, USA; o Uniformed Services University, Bethesda, MD 120814, USA

**Keywords:** Enteric diseases, Vaccines, PDVAC, Burden, Modelling

## Abstract

Investment in vaccine product development should be guided by up-to-date and transparent global burden of disease estimates, which are also fundamental to policy recommendation and vaccine introduction decisions. For low- and middle-income countries (LMICs), vaccine prioritization is primarily driven by the number of deaths caused by different pathogens. Enteric diseases are known to be a major cause of death in LMICs. The two main modelling groups providing mortality estimates for enteric diseases are the Institute for Health Metrics and Evaluation (IHME) at the University of Washington, Seattle and the Maternal Child Epidemiology Estimation (MCEE) group, led by Johns Hopkins Bloomberg School of Public Health. Whilst previous global diarrhoea mortality estimates for under five-year-olds from these two groups were closely aligned, more recent estimates for 2016 have diverged, particularly with respect to numbers of deaths attributable to different enteric pathogens. This has impacted prioritization and investment decisions for vaccines in the development pipeline.

The mission of the Product Development for Vaccines Advisory Committee (PDVAC) at the World Health Organisation (WHO) is to accelerate product development of vaccines and technologies that are urgently needed and ensure they are appropriately targeted for use in LMICs. At their 2018 meeting, PDVAC recommended the formation of an independent working group of subject matter experts to explore the reasons for the difference between the IHME and MCEE estimates, and to assess the respective strengths and limitations of the estimation approaches adopted, including a review of the data on which the estimates are based.

Here, we report on the proceedings and recommendations from a consultation with the working group of experts, the IHME and MCEE modelling groups, and other key stakeholders. We briefly review the methodological approaches of both groups and provide a series of proposals for investigating the drivers for the differences in enteric disease burden estimates.

## 1 Background

Whilst it is estimated that diarrhoeal mortality has decreased by more than 20% from the decade between 2005 and 2015, the burden of diarrhoea is still significant and predominantly affects sub-Saharan Africa and South Asia in populations with poor access to primary healthcare, clean water and sanitation [1].

Today, diarrhoeal diseases with the highest burden in under five-year olds are considered to be rotavirus, *Shigella* and *Salmonella* species. *Cryptosporidium* and enterotoxigenic *Escherichia coli* (ETEC) also contribute significantly to overall burden [1]. In 1975, the WHO recommended oral rehydration solution globally as the standard immediate treatment for acute diarrhea [2], yet recent evidence suggests that recognition by caregivers may be poor, and uptake remains low [3]. The routine use of antimicrobials for diarrhoea in children is only recommended by WHO in clinically severe cases for cholera, shigellosis, dysenteric presentation of campylobacteriosis and non-typhoidal salmonellosis, or when the host immune status is severely compromised by severe malnutrition or chronic disease [4]. However, with the recognition and continued emergence of antimicrobial resistance (AMR) [5], particularly amongst diarrhoea-related pathogens [6], [7], additional approaches to tackling childhood diarrhoea, particularly in LMICs, must be sought. Future research and development of vaccines against diarrhoeal pathogens have been highlighted as key priorities to reduce global disease burden [8], [9]. There are currently licensed vaccines for rotavirus and cholera; however, vaccines are not currently available for any of the other major diarrhoeal pathogens, although all have candidates in clinical development.

The estimated global burden of disease, and in-particular the mortality attributable to each pathogen, impacts priority setting for investment in vaccine research and development (R&D), as well as policy recommendations for introduction of new vaccines into immunization programmes. It is important, therefore, that the methodology used to derive the estimates is well understood, and accepted, by global stakeholders, including product developers and policy-makers. Two modelling groups, IHME and MCEE, have generated estimates of mortality due to the different causes of diarrhoea. Historically, the global diarrhoea mortality estimates for children under 5 years of age (U5s) from both groups have been similar with broad and overlapping confidence intervals [10], [11], [12]. However, the most recent iteration of estimates from IHME, GBD 2017 for the year 2016, have diverged from previous IHME estimates, particularly with respect to the numbers of deaths attributed to specific pathogens, mainly as a result of revisions to the estimates in high-population, high-burden countries. With the adoption of new detection technology for diarrhoeal pathogens in stools and changes to the methodological approach, estimates of the mortality associated with some pathogens has shifted so significantly that investment in some vaccine candidates that are approaching late-stage clinical testing has been reduced[13]. Because of the implications of changes in disease burden estimates, in 2018, WHO’s Product Development for Vaccines Advisory Committee (PDVAC) recommended that an independent working group be established to evaluate diarrhoeal burden models and estimates [14].

The enteric burden of disease working group (WG) was established and convened in 2018. The initial focus of the WG has been on the aetiology of diarrhoeal deaths in children U5. Morbidity and long-term sequelae, though constituting a significant proportion of the burden from enteric pathogens in this age group, have not yet been considered by the WG, nor has mortality in older age groups. However, these factors need to be included in the assessment of the global burden of various diarrhoeal pathogens and the potential public health value that a vaccine could offer.

This report summarizes the findings from the first meeting of the WG.

## 2 Objectives of the workshop

The IHME GBD and the MCEE models were reviewed with the objectives to:

(I) Identify areas of commonality and divergence across methodologies and assumptions.
(II) Develop recommendations and identify areas for further work that may inform future iterations of the diarrhoeal aetiology-specific mortality assessments for U5s.
(III) Increase the transparency and understanding of how aetiology estimates are derived.

The sections below outline the main methodological approaches of the two modelling groups, highlighting similarities and differences. We use *Shigella* and ETEC as examples to show the divergence in modelled estimates between the groups. Reference will be made to two large landmark epidemiology studies, namely the Global Enteric Multicentre Study of Diarrheal Disease (GEMS) [15] and the Aetiology, Risk Factors, and Interactions of Enteric Infections and Malnutrition and the Consequences for Child Health and Development study (MAL-ED) [16] (see Box 1). Finally, a set of proposals are presented as future work to explore variations in the model outputs and to provide updated evidence for key assumptions applied by both modelling groups.

Box 1 **Description of two large landmark epidemiology studies of diarrhoeal disease aetiology, GEMS and MAL-ED.**

**The Global Enteric Multicentre Study of Diarrheal Disease (GEMS)**

The GEMS study was a 3-year, prospective, age-stratified, case-control study to estimate the population burden and microbiologic aetiology of acute moderate-to-severe diarrhoea (MSD). Children aged 0–59 months seeking care for diarrhoea as outpatients or inpatients at health care centres (cases) were compared with non-diarrhoeal community controls. In addition, cases were followed up after 2 months to study short-term mortality after an episode of MSD. The research was carried out in seven field sites in Southern Asia and sub-Saharan Africa, using qPCR as a diagnostic tool in cases and controls to detect evidence of different pathogens in stool samples and these results were used to estimate the fraction of moderate-to-severe diarrhoea attributable to each pathogen.

**The Aetiology, Risk Factors, and Interactions of Enteric Infections and Malnutrition and the Consequences for Child Health and Development (MAL-ED)**

The MAL-ED study was a birth cohort study at eight sites in South America, sub-Saharan Africa, and Asia. It used a prospective longitudinal design to assess diarrhoea, subclinical enteropathogen carriage and undernutrition up to the age of 2 years in a community setting. Among the factors evaluated were enteric infections (with or without diarrhoea) and other indicators including micronutrient levels, diet, socioeconomic status, gut function and environment. The study examined these factors, their inter-relationships and overall impact on health outcomes. Because participants were selected in the community, rather than from among hospital patients, few children with severe, dehydrating diarrhoea were observed.

## 3 Overview: modelling estimates of mortality due to enteric infections

**Fig. 1:**
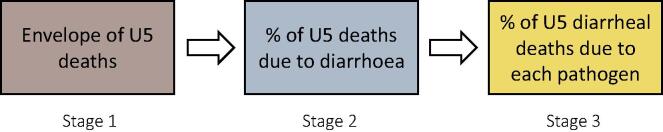
Three stage process for generating U5 diarrheal deaths due to different pathogens.

Both the IHME and MCEE modelling groups employ a step-wise process for generating under-five all-cause and pathogen-specific diarrhoea mortality estimates. The first stage is to generate estimates for all-cause mortality in U5s. This is then disaggregated into the percentage of U5 deaths due to diarrhoea, and then, estimates for deaths attributed to individual pathogens are generated (Fig. 1). Table 1 compares differences in these first two key outputs from the two modelling groups and provides information on differences in data sources and inclusion and exclusion criteria applied to generate the estimates. In addition, estimates for ETEC and Shigella are provided, comparing 2011 estimates for MCEE and 2013 for IHME from earlier studies, as an example of deaths attributed to different pathogens during the third stage. Data from IHME 2016 are also shown, data for MCEE 2016 will not be made publicly available. In this section, we outline the first two stages shown in Fig. 1 and then provide a more detailed overview of the third stage employed by each group to generate estimates of the numbers of diarrhoeal deaths attributable to different aetiologies, highlighting key similarities and differences in Section 4.

**Table 1:** Differences in data sources used and estimates generated for the envelope of U5 deaths and percentage of U5 deaths due to diarrhoea, by MCEE and IHME. A comparison for the percentage of U5 diarrheal deaths due to Shigella and ETEC are provided as examples to show the variation at the level of aetiology.

Model output	IHME (2016)^1^	MCEE (2016)^2^
U5 Mortality Envelope (2017) (Stage 1)	IHME-generated estimates	Generated by the United Nations Group for Child Mortality Estimates (UN IGME)
	5·6M (5·4M-5·9M)	5·4M (5·2M-5·8M)

U5 Deaths Due to Diarrhoea (Stage 2)	Data included: • Vital registration studies with >60% data completeness. • Verbal autopsy data from demographic surveillance and surveys	Data included: • Vital registration studies with >80% data completeness. • Verbal autopsy data from demographic surveillance and surveys.
	549 K (491–606 K)	477 K (375–555 K)

U5 Diarrheal Deaths Due to Pathogens: (Stage3) Shigella: ETEC:	• All hospital and community studies conducted for 12 or more months. • GEMS and MAL-ED studies.	• Hospital inpatient studies conducted for 12 or more months. • GEMS and MAL-ED unpublished studies including data stratified by inpatient vs. outpatient/community.

Model output	IHME (2016)	MCEE (2016)
Shigella	99 680 (59550–161235)	Data will not be made publicly available
ETEC	15 960 (4400–40300)	Data will not be made publicly available
	**IHME (2011)**^3^	**MCEE (2013)**^4^

Shigella	33 400 (24 900–43500)	28 000 (12000–53000)
ETEC	23 100 (17000–30400)	42 000 (20000–76000)

Comparison of modelling results and data inputs for IHME and MCEE for Total U5 mortality envelope (2017) and percentage U5 deaths due to diarrhoea (2017). Estimates for percentage U5 diarrheal deaths due to Shigella and ETEC (2016 GBD IHME, 2017 MCEE (unpublished)) are also shown.

1 Data extracted from GHDx website for the Global Burden of Disease 2017 Model, reporting data for 2016.

2 Data from Pneumonia and Diarrhea Progress Report, 2018. John Hopkins, International Vaccine Access Centre.

3 Data from Global, regional, and national age-sex specific all-cause and cause-specific mortality for 240 causes of death, 1990–2013: a systematic analysis for the Global Burden of Disease Study 2013. Lancet 2015, Jan 10; 385(9963): 117–171.

4 Data from Global Causes of Diarrheal Disease Mortality in Children <5 Years of Age: A Systematic Review. PLOS One 2013, 4 Sept. 8(9).

### 3.1 Generation of the total global envelope for all-cause U5 mortality globally

For the 2017 iteration, the IHME model uses IHME-generated estimates for all demographic inputs. The MCEE model uses United Nations Inter-agency Group for Child Mortality Estimation (UN IGME) modelled estimates, generated using UN Population Division demographic data of population, fertility, migration and mortality by age, sex, location and time, from national censuses and specialised surveys [17]. Despite these differences in the source data, the overall U5 mortality estimates generated by each group are similar; 5·6M (95%UI, 5·4M-5·9M) for IHME and 5·4M (90%UI, 5·2M-5·8M) for MCEE. However, it should be noted that there are significant differences between the mortality estimates in different geographic locations. Thus, differences in the all-cause envelope at a country or regional level, used by the IHME modellers, could lead to differences in the estimated number of total diarrhoea deaths for individual regions between IHME and MCEE. For each revision of the GBD estimates, both groups update all of their values for previous years.

The second output is an estimate for the percentage of U5 deaths due to diarrhoea. Both IHME and MCEE use vital registration, verbal autopsy and surveillance system data to inform the estimate of percentage of U5 deaths due to diarrhoea. However, there are differences in the data used by each of the groups, with respect to inclusion and exclusion criteria and access to unpublished data. The MCEE group have more stringent inclusion and exclusion criteria for the threshold completeness of vital registration data: MCEE only accepts studies with >80% data complete whereas IHME includes studies with > 60% data completeness.

To generate diarrhoeal-specific estimates, IHME redistributes deaths that cannot be atrtibuted to a pathogen to specific causes of death (like diarrhoea) and uses an internal model called the Cause of Death Ensemble model (CODEm), a Bayesian hierarchical model to generate, by location, age group (four age groups under 5 years), sex, and year, estimates of the percentage of deaths due to diarrhoea. Table 1 provides information on the data sources used for the first two stages in the process.

The procedures used by both groups to generate the percentage of U5 diarrheal deaths due to different pathogens, which was the major focus of the workshop. Differences in methodological approaches between groups at this stage are most likely to contribute to the divergence seen in pathogen-specific mortality estimates (aetiology). In Section 4, each step in the process is summarised and an explanation of similarities and differences in the modelling approaches are reviewed, highlighting the strengths and limitations of the methodologies used by each group.

## 4 Process for calculating the percentage of diarrheal deaths due to individual pathogens

### 4.1 Generation of a diarrheal mortality proxy

Due to a lack of representative datasets in which deaths due to specific diarrhoeal pathogens have been identified, the initial step in the process of attributing diarrhoeal deaths to specific pathogens is to identify a proxy measure. Both groups use the proportion of diarrheal episodes in which a particular pathogen is detected as the proxy measure; however, IHME use data from both hospital inpatient and outpatient studies and MCEE uses only data from hospital inpatient studies, assuming that hospitalization is a more accurate proxy for mortality and that aetiologic fractions differ between inpatient and outpatient studies. Both groups only use studies which were conducted for 12 or more months. Studies which did not stratify data from inpatients, outpatients and the community were excluded by MCEE. Facility-based studies of both inpatients and outpatients are thus only included if inpatient-specific proportions are reported or raw data are available to allow subset analysis with the exception of the GEMS and MAL-ED studies, for which unpublished stratified data were obtained. Both IHME and MCEE include only diarrhoea proportion data from studies with a minimum of 100 samples tested and conducted for longer than one year, and not restricted to specific subpopulations. The proportion positive in hospitalised and ‘severe’ episodes are tracked, and all data meeting inclusion criteria is used in the models [18]. All data for hospitalised/severe diarrhoea relative to community/outpatient are included in the model, including all eligible GEMS data. A summary is given in Table 2.

**Table 2:** Summary of data used by IHME and MCEE for generating diarrheal mortality proxy.

Model Methodology	IHME	MCEE
Generation of diarrheal mortality proxy	Include hospitalised and ‘severe’ diarrheal episodes.	Included only hospitalised episodes.
	Include inpatient, outpatient, and community-based data.	Include only inpatient data.
	All eligible GEMS and MAL-ED data included.	Only GEMS and MAL-ED inpatient data included

#### 4.1.1 Strengths and limitations

The approach of IHME is to use all available data with limited restrictions, whilst MCEE have stringent inclusion and exclusion criteria for data. The inclusion of ‘severe’ cases as well as hospitalised cases allows both inpatient and outpatient data to be used for IHME’s model estimates. However, there is variation between studies in what may constitute a ‘severe’ case (i.e. the case definition), and the interpretation of this, which may limit data quality. The IHME approach uses more data compared to MCEE, although there is likely to be a large proportion of studies that were included by both modelling groups. A comparative analysis of the criteria for data inclusion, data sources and data quality of the modelling groups could help to optimize approaches for the next iterations of models, ensuring that future diarrhoeal disease models abide to a high and consistent standard of data quality.

### 4.2 Validity of epidemiology studies to be included

Both groups use systematic literature reviews that date back to 1985 (IHME) or 1990 (MCEE), to extract pathogen prevalence from studies meeting their inclusion and exclusion criteria. This includes, where available, data from scientific literature on different aetiologies, hospital data, population survey data, and data from unpublished sources, including detailed data for the GEMS and MAL-ED studies and more recent cohort studies.

#### 4.2.1 Strengths and limitations

The validity of using studies dating back to the 1980's by both groups, in terms of robustness and reproducibility of diagnostic methods compared to more recent studies, is highlighted as a potential limitation. The improvement over time in culture-based and other diagnostic techniques as well as likely shifts in the aetiology of diarrhoeal disease, due to initiatives such as Water, Sanitation and Hygiene (WASH), may suggest that, for contemporary estimates, a greater weighting should be placed on more recent studies [19]. Additionally, evidence from GEMS shows that using qPCR to test stools for different pathogens is more sensitive than culture-based methods, compared to culture-based methods [20], with the consequence that older diagnostic reporting from older studies may underestimate the prevalence of pathogens which require more sensitive techniques for detection. There is a need for sensitivity analysis to test for reliability of these data, and groups should consider the potential effect of attributing greater weighting to more recent studies or incorporating time and/or time-varying covariates in the models [21], given the improvement in diagnostics.

### 4.3 Data processing methodologies and the use of qPCR data

#### 4.3.1 IHME approach

The final step in the generation of estimates for individual pathogen mortality is data adjustment and processing. In the case of IHME, epidemiological data extracted from the literature, hospital data, and population survey data are analysed using their DisMod tool (Bayesian hierarchical meta-regression tool) to estimate the proportion of diarrhoeal cases that are caused by different organisms. In order to adjust for the different pathogen detection methods used, GEMS and MAL-ED data are used to calculate the sensitivity and specificity of laboratory diagnostic tests, such as culture and immunoassay, relative to qPCR, for each pathogen and a qPCR adjustment factor is applied to the pathogen-specific proportions after the model is run. A major short-coming of diarrheal disease studies conducted prior to GEMS has been the failure to perform comprehensive assessment of major enteric pathogens, due to demanding technical approaches, in settings with high burdens, often lacking the necessary laboratories and diagnostics [22]. The correction factor generated from GEMS and MAL-ED is then applied to the modelled results and adjusted for sensitivity and specificity based on this estimate. The modelled proportion of diarrhoeal cases by aetiology are adjusted and produce estimates for each location, year, age, and sex. IHME then use a counterfactual approach to calculate a population attributable fraction (PAF) for each pathogen, which represents the relative reduction in hospitalised diarrhoea episodes (as a proxy for deaths) if exposure to a given aetiology was to be eliminated. In order to generate this, GEMS case-control data are used to calculate pathogen-specific odds ratios (ORs) by age where the OR is the odds of diarrhoea given the presence of that pathogen in the stool at a diarrhoea-attributable quantity as detected by qPCR, divided by the odds of diarrhoea without the detection of the pathogen at a diarrhoea-attributable quantity. This is combined with the qPCR-adjusted proportion of diarrhoea cases positive for a given aetiology to calculate the pathogen-specific PAF. Such that:

$$\mathrm{PAF}=Proportion*\left(1-\frac{1}{\mathrm{OR}}\right)$$

**Fig. 2:**
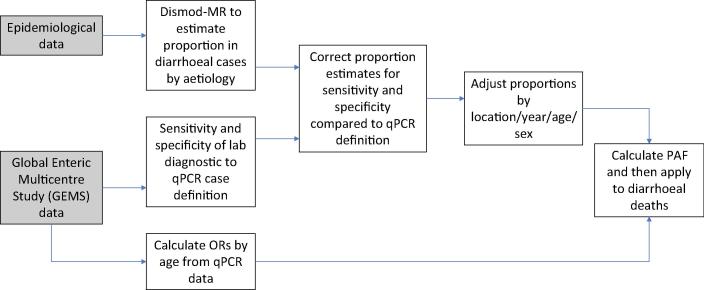
Data processing mechanism for IHME data (adapted from GBD Diarrhoeal Diseases Collaborators. Estimates of global, regional, and national morbidity, mortality, and aetiologies of diarrhoeal diseases: a systematic analysis for the Global Burden of Disease Study 2015. Lancet Infect Dis 2017).

The PAF is applied to calculate deaths due to diarrheal aetiologies by location, year, age, and sex. The schematic in Fig. 2, below, summarises the IHME data processing methodology.

For all pathogen aetiologies except *Vibro cholerae* and *Clostridium difficile*, the PAF is calculated from the proportion of diarrhoea cases that test positive for each aetiology and the OR of having diarrhoea if the pathogen is detected. Since diarrhoea can be caused by co-infections with multiple pathogens, PAFs can overlap and therefore add up to more than 1. The purpose of using ORs is to address the issue of subclinical carriage, namely that enteric pathogens are frequently detected in the absence of diarrhoea. For each pathogen, PAFs and uncertainty intervals are multiplied by the diarrhoea mortality envelopes to estimate age-, sex-, location- and year-specific pathogen deaths.

#### 4.3.2 MCEE approach

**Fig. 3:**
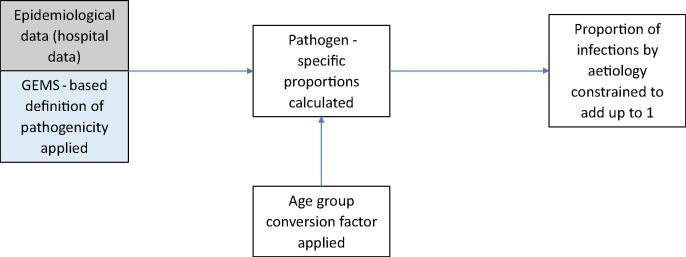
Data processing mechanism for MCEE data.

MCEE takes a different approach. They do not adjust studies and data where pathogens are detected using non-qPCR detection methods to match with those detected by qPCR. However, MCEE uses pathogen positivity according to GEMS qPCR to define pathogenicity. Organisms with <2 site- and age-specific attributable fractions that were different to 1 (not associated with causing diarrhoea) in the GEMS qPCR re-analysis [20] (including norovirus G1, atypical EPEC, LT-ETEC, Giardia, EAEC) were excluded from the analysis. Mean age-adjusted pathogen-specific proportions were calculated from hospital data, with age-group conversion factors applied to convert pathogen-specific proportions reported for narrower age ranges than 0–59 months, based on data from studies that reported estimates across age ranges. A global median was used to estimate pathogen-specific proportions for regions with missing data. The pathogen proportions (in addition to the unknown proportion, derived from GEMS qPCR hospital inpatient data) are constrained to add up to 1 overall and by region (Fig. 3). Uncertainty bounds are generated using bootstrap techniques. Final pathogen-specific proportions and uncertainty intervals are multiplied by regional diarrhoea mortality envelopes by year to generate regional estimates of pathogen-specific deaths.

#### 4.3.3 Strengths and limitations

Samples from the GEMS study have recently been reanalysed using qPCR methods rather than the culture-based and other microbiological methods that were used in the original study. This has led to a re-estimation of the percentage of diarrhoea attributable to a named pathogen, with 89% of all episodes attributed to a pathogen, compared to 52% in the original GEMS analysis [20]. In the modelling analysis, IHME uses these reanalysed GEMS data to adjust for differences in sensitivity and specificity for all PCR and non-PCR estimates, whilst MCEE does not, but plans to do so in the future. There are two main limitations in using the GEMS data. Firstly, the use of a single adjustment for each pathogen derived from GEMS ignores substantial heterogeneity across studies in the relative sensitivity between diagnostics. Secondly, the absence of diagnostic metadata in the IHME database combined with the increasing incorporation of molecular diagnostics may lead to over-correction, particularly among more recent studies that use PCR.

### 4.4 Approaches to infer aetiology in the presence of high-rates of subclinical pathogen detection

#### 4.4.1 The use of PAF versus pathogen prevalence

Currently, only IHME uses ORs to calculate the pathogen-specific mortality and population attributable risk, whilst MCEE use the pathogen prevalence (the prevalence of individual pathogens from the studies). Both groups consider evidence from multiple pathogen studies, thereby addressing the issue of mixed aetiologies. This gives rise to the issue of subclinical carriage, where a pathogen may be present but is not responsible for symptoms. This approach relies heavily on the assumption that the ratio of the prevalence of a pathogen in cases compared to controls is a clear indicator of pathogenicity. This assumption may be too conservative, especially in high incidence settings [23], [24] and in-particular for pathogens that commonly cause reinfection. Such is the case for many enteric pathogens that often cause asymptomatic or subclinical infection.

IHME does not constrain the PAFs to add up to 1, and therefore they assume that in instances where multiple pathogens are detected, diarrhoea would still occur even if one of the pathogens was removed. MCEE also considers mortality attribution may be a result of multiple pathogens, but they do constrain the pathogen prevalence envelope to add up to 1 minus the proportion of diarrhoea without a known cause (restricting to the inclusion of pathogens that were statistically significantly associated with diarrhoea in the GEMS qPCR re-analysis). Burden estimates could be significantly inflated, if subclinical carriage (presence of a pathogen, which is not severe enough to present observable symptoms) is not considered, by way of calculating attributable fractions. In addition to this there may be variation in pathogen load needed to cause infection between sites, i.e. variations in the probability of clinical symptoms caused by a certain pathogen in different settings, which would lead to a restriction in the generalizability of assumptions. It therefore remains unclear how often ‘true’ aetiologic mixed infections occur. Evidence from qPCR shows high variation in durations of carriage of subclinical infections, and there is also evidence to suggest infections could be sequential rather than mixed [25], [26]. The implications of this are that, in cases where a pathogen is consistently shed and there are high levels of detection but suspected low levels of pathogenicity within a site, country, or region, the OR may be over- or under-estimated.

#### 4.4.2 Strengths and limitations

A strength of the current approach of both groups is the consideration of sub-clinical infections, but there is a lack of knowledge on how to interpret the information and estimates of the burden of sub-clinical infections which are not generated by either group. The presence of sub-clinical infection may vary geographically for individual pathogens, such that the occurrence of one pathogen may cause symptoms in one setting but not in another. A further limitation is lack of knowledge on whether co-infections enhance the probability of clinical symptoms and how different pathogens interact and impact aetiology in the presence of others. This is potentially further complicated by geographical variations and a lack of understanding on whether infections are ‘mixed’ or ‘sequential’. For IHME, these combined factors may result in heterogeneity of ORs that complicates the use of a single ‘global’ median OR for each pathogen to calculate PAFs, currently adopted by IHME. A potential solution, to strengthen the approach adopted by IHME, may be to generate ORs across similar geographical regions and population archetypes, based on the proportion of pathogens excreted in diarrheal and non-diarrheal stools. However, the sparsity of data on pathogen prevalence that is currently available from healthy controls limits this option, and additional control samples from geographical areas would need to be generated. This is also a limitation for the MCEE group, who considers that mortality may be attributed to multiple pathogens, with pathogen prevalence informing aetiology. Additional information on pathogen prevalence in healthy controls, would likewise help to also strengthen MCEE’s approach.

### 4.5 Other key strengths and limitations in the generation of individual pathogen estimates

#### 4.5.1 Data extrapolation

In order, to produce global estimates for individual pathogen estimates, IHME extrapolate the ORs derived from children under 5 years of age from Africa and Asia (GEMS) to all estimates, including adults and in developed countries. Prevalence of each pathogen is estimated for both ages and locations where data do not currently exist, and models are continually updated. Whilst in many instances, sub-regional data may be able to predict country-level burden attribution with a high degree of confidence, it is possible that outliers, where data quality is poor or is not reflective of sub-regional estimates, may result in inaccurate estimates for some countries. MCEE use a global median to estimate pathogen-specific proportions for regions with missing data. This is reliant on the data being a good proxy for other regional estimates, and it is not clear if this is always the case.

#### 4.5.2 Assumption of pathogen homogeneity in case fatality ratios

Both modelling approaches assume that the distribution of pathogen aetiology as assessed via each group’s accepted proxy measure directly reflects the distribution of mortality, such that detection of every pathogen in a hospitalized or severely ill child is assumed to be equally likely to cause death. MCEE assume that case fatality ratios are the same across all pathogens based on the prevalence estimate they generate when they scale pathogen-specific proportions. The same implicit assumption is made by IHME, when they generate PAFs. If indeed, there is variation between pathogens, this will affect the distribution of pathogen aetiology in patients who die from diarrhoea and may also be dependent on geography, socio-economic status and population. More evidence is needed to explore variations in case-fatality rates for individual pathogens. This is challenging because the majority of data on mortality is obtained in hospital settings and therefore may bias estimates, given that untreated severe diarrhoea within the community, particularly in LMICs, is more likely to result in death, in most cases. Data collected from study sites is also extrapolated at country-level, which may not necessarily be representative. Whilst this is a limitation, to address this issue of generalisability would be particularly difficult.

## 5 Proposals for addressing knowledge gaps and improving model estimates

There are both strengths and limitations to the methods presented by both IHME and MCEE groups, as outlined above. We believe that a key outcome of the consultation should be to initially focus on understanding the main reasons for variations in aetiology estimates within the published diarrhoea mortality envelope for U5s. Three key thematic areas were proposed for future investigation.

(1) How differences in data inputs impact the distribution of U5 aetiology estimates within the mortality envelope.

Identifying the drivers for differences between the modelling outputs produced by IHME and MCEE will be a key step in helping to quantify and assess the source of differences in estimates. Whilst much of the data used by both groups are expected to be common and comparable, there are important differences in the criteria that determine which data are included. An improved understanding of factors that are the strongest determinants of the model estimates is vital to better understanding their accuracy and robustness, since these estimates drive decision-making with respect to vaccine development and introduction. The impact or trade-off of utilising a small number of high-quality studies (as MCEE do) versus a larger number of studies of varying quality (as IHME do), to generate global estimates needs to be investigated. Additionally, the validity of using historical studies that use less sensitive diagnostic techniques, weighted equally within the data analysis, needs reviewing.

To investigate these steps, we propose the following strategies, to be refined in an iterative manner as greater understanding of the processes evolve:

i. Conduct a systematic comparison of studies and data included in both models, in an effort to understand how data selection criteria and access to data impacts on the quality and quantity of studies used.
ii. Agree on a standardised dataset to directly compare how differences in model structure and associated assumptions (rather than input data) impact estimates.
iii. Conduct a data grading review to improve understanding of the quality and standard of data used by each group, followed by a model sensitivity analysis to explore the impact of inclusion and exclusion criteria on model estimates.
(2) How extrapolation of odds ratios from 7 GEMS sites in Africa and Asia impacts global aetiology estimates.

There were concerns regarding potential bias resulting from the use of GEMS data to determine global median ORs or pathogens, given substantial variation in the rates of subclinical pathogen carriage across ages and settings. Concerns were further raised regarding the direct use of odds ratios exclusively from seven GEMS sites and extrapolation of this data regionally and to older age groups (5–99 years, for Global Burden of Disease). There was a desire to improve our understanding of the relationship between the presence of pathogen and diarrhoea by utilising additional data from controls to provide more geographically representative ORs which can be consistently used in future modelling analysis, for both IHME, MCEE and other groups. Recommendations included:

i. Conduct a systematic review to identify studies that tested for the presence of key diarrheal pathogens among non-diarrheal controls and older age groups.
ii. Use the findings to carry out a careful and comprehensive statistical approach using network meta-analysis to assess variability in odds ratios within and across regions and settings, built through consensus with IHME and MCEE.
(3) Heterogeneity in case fatality ratios (CFRs) for different pathogens.

There is a strong recommendation to assess potential variability in CFRs, particularly in-light of the high number of co-infections and sub-clinical carriage reported in study participants in low- and middle-income countries, and to assess the relationship between the OR of disease and risk of death. This approach will need to account for the different approaches taken by each of the modelling groups, since neither group uses CFR in its current methodology, to ensure the process provides useful information that can be adapted and applied by both MCEE and IHME in the future. A limitation is the potential scarcity of data available for non-hospitalised (community) estimates of death (as previously alluded to), which will likely introduce bias since treatment within a hospital setting is likely to significantly increase chances of survival. Additionally, mortality may be affected by the rate of onset of illness and time to death (which may affect the probability that a child with diarrhoea reaches a facility), and this may vary depending on the pathogen. Factors such as economic status and geography are also important. To address this issue, there was a recommendation to:

i. Carry out a systematic literature review to identify the CFR for selected enteric pathogens. The review should aim to include both hospital and non-hospital data (with the caveat that the latter may be very limited; and that hospitalization changes the CFR for largely treatable infections, like rotavirus.).

## 6 Discussion

The global mortality due to diarrhoeal disease is declining, and for some pathogens, such as ETEC, the current (GBD 2017 for the year 2016) IHME burden of disease estimate is now considered too low to warrant prioritization of vaccine development by some stakeholders. However, given the variation between mortality estimates from different groups, the methodology and robustness of each estimate needs to be carefully understood and investigated. For this reason, WHO’s PDVAC recommended the formation of an independent working group to evaluate these aspects. As we have discussed here, we identified several elements of the methodology that we suggest, be further investigated.

The proposed systematic review to extract data from healthy controls will help to further explore the relationship between the presence of a pathogen and disease. It is expected there will be a degree of geographical variation within the results, to complement the current GEMS data, which assumes homogeneity across sites for the odds ratios. This additional information may help to better explain geographical differences in the susceptibility of individuals and populations to certain pathogens, and thus provide improved information on burden.

The results from the second systematic review will explore the likelihood of mortality given the presence of disease, known as the case fatality ratio, for individual pathogens. The current strategy employed by both modelling groups is to assume equal likelihood of death regardless of the aetiology. This is likely to over-estimate the risk of mortality for some pathogens and under-estimate the risk for others. A clearer understanding of the variation in case fatality ratio will be of great importance in informing vaccine development, particularly for pathogens which are associated with a higher risk of mortality.

## 7 Conclusion

The modelling approaches employed by both the IHME and MCEE groups have historically provided well-regarded results that have guided policy recommendations. As new data, diagnostic and surveillance techniques emerge, the incorporation of new evidence is essential to ensure more reliable estimates. However, the interpretation and use of new evidence should be subject to a high level of scrutiny, and where appropriate, new guidelines developed for its use. In addition, it will be important to address gaps within the data that give rise to inconsistencies and less reliable information. Emerging evidence generated through studies, such as The Child Health and Mortality Prevention Surveillance (CHAMPS) study, designed to track definitive causes of child mortality in sites throughout Asia and sub-Saharan Africa through minimally invasive tissue sampling, will help to provide validation on key measures such as the diarrhoeal mortality proxy. More generally, steps can be taken to assess the quality of data employed by modelling groups, and systematic reviews can be undertaken to address other key gaps in the evidence.

Future modelling work, should also assess the longer-term sequalae of diarrhea [27], [28], including growth failure and cognitive impairment in the earlier years of life, as well as metabolic syndrome in the later years of life [29]. There is evidence also that repeated infections may increase risk of death from other unrelated infectious diseases such as pneumonia and malaria [30]. Whilst the GBD estimates include growth deficits in their estimation of diarrhoea-specific DALYs, they do not consider neurodevelopment due to the absences of standard routinely collected metrics of neurodevelopment. Future models should incorporate these longer-term effects and risk factors, in addition to the effects of acute diarrhoea. This will further strengthen the evidence-base for decision-making around prioritizing enteric pathogens for which vaccines would have the greatest public health impact.

There is a clear case for ensuring diarrhoeal pathogen mortality estimates are constantly reviewed and updated, and adequate resources are made available to ensure model comparison exercises, since these are a core component of vaccine priority evaluations. This work will serve as an example of the importance of critically assessing data quality in modelling exercises, whilst identifying and addressing crucial gaps in the evidence which must be addressed.

The proposed work aims to provide a thorough assessment of the current data and approaches used by IHME and MCEE groups. Our hope is that this can include a sensitivity analysis of the model input data to improve understanding of key input variables, potentially leading to improved guidance for data use, and enhanced interpretation of model results to guide decision-making. Whilst the activities outlined above help to better define which pathogens should be prioritized for vaccine development, future work should include assessment of data and model to quantify the impact of longer-term sequelae. Collectively, robust data and burden models are imperative for building the evidence-base to inform prioritization and policy decisions.

## 8 Role of the funders

We gratefully acknowledge funding from 10.13039/100000865Bill & Melinda Gates Foundation for funding support (OPP1135836). The views, findings, and conclusions contained within are those of the authors and should not be construed to represent the positions or policies of the Bill & Melinda Gates Foundation, the US Department of Defense, or the World Health Organisation. We are very grateful to all participants at the meeting.

## Authors contributions

HJ Prudden and B Giersing contributed significantly to the development of text and content, produced the main figures and tables and carried out extensive editing. RE Black, C Troeger, and RC Reiner contributed to the development of the methodology and details of the overview (3) section of the paper, as well as additional edits to the main manuscript.

M Hasso-Agopsowicz, R Breiman, M Jit, G Kang, L Lamberti, CF Lanata, BA Lopman, W Ndifon, VE Pitzer, J Platts-Mills, M Riddle, P Smith, and R Hutubessy, as members of the WHO Burden of Disease Working Group, all contributed to multiple rounds of editing of the document, the production of figures and the table, and the overall structure of the manuscript. We declare that all authors have reviewed and approved the final article.
